# Erroneous or Arrhenius: A Degradation Rate-Based Model for EPDM during Homogeneous Ageing

**DOI:** 10.3390/polym12092152

**Published:** 2020-09-21

**Authors:** Maha Zaghdoudi, Anja Kömmling, Matthias Jaunich, Dietmar Wolff

**Affiliations:** Bundesanstalt für Materialforschung und -prüfung (BAM), 12200 Berlin, Germany; anja.koemmling@bam.de (A.K.); matthias.jaunich@bam.de (M.J.); dietmar.wolff@bam.de (D.W.)

**Keywords:** kinetic study, Arrhenius, TTS, modeling, chemical processes, stress relaxation

## Abstract

To improve the predictive capability of long-term stress relaxation of elastomers during thermo-oxidative ageing, a method to separate reversible and irreversible processes was adopted. The separation is performed through the analysis of compression set after tempering. On the basis of this separation, a numerical model for long-term stress relaxation during homogeneous ageing is proposed. The model consists of an additive contribution of physical and chemical relaxation. Computer simulations of compression stress relaxation were performed for long ageing times and the results were validated with the Arrhenius treatment, the kinetic study and the time-temperature superposition technique based on experimental data. For chemical relaxation, two decay functions are introduced each with an activation energy and a degradative process. The first process with the lower activation energy dominates at lower ageing times, while the second one with the higher activation energy at longer ageing times. A degradation-rate based model for the evolution of each process and its contribution to the total system during homogeneous ageing is proposed. The main advantage of the model is the possibility to quickly validate the interpolation at lower temperatures within the range of slower chemical processes without forcing a straight-line extrapolation.

## 1. Introduction

Lifetime prediction of elastomer components is a very challenging task due to many factors. The first factor is the variety of fields of application and their requirements ranging from coatings in medical application [1] to isolation bearings in construction [2] or even to special sealing applications such as e.g., in containers designed for transport, storage and/or disposal of radioactive materials. The second factor are the environmental conditions to which the elastomer components are exposed during application. Environmental conditions are for instance moisture, weathering, oxygen, mechanical loading, radiation (e.g., γ-radiation in nuclear application [3]), contact with chemical (e.g., in vehicle engine exposed to different fuels [4]) or biological media and temperature, which can be encountered separately or in combination. Probably the most common combined environmental conditions are oxygen, mechanical loading and temperature. When elastomers are exposed to these conditions, their functionality in operation might be limited due to degradation. Degradation due to ageing is caused by both physical and chemical processes [5]. Physical ageing is mostly a reversible process, and is often associated with macromolecular rearrangement [6]. Chemical ageing, on the other hand, is an irreversible process that leads to oxidation, chain scission, and/or the formation of new cross-links [5]. These irreversible microstructural changes are thermally driven effects and affect the mechanical properties of the material. Thus, oxidative ageing plays a crucial role in limiting the lifetime of elastomer components. Typically, the change of a chosen mechanical property is evaluated under accelerated ageing conditions, in order to extrapolate it to a suitable lower temperature such as e.g., room temperature. Tensile and compressive stress–relaxation methods have often been used to monitor and predict seal material lifetimes, as the relaxation process has an influence on the seal performance [7]. Lifetime predictions are based on the determination of a degradation activation energy which quantifies the relationship between temperature and the rate of degradation. The Arrhenius approach is commonly used in lifetime prediction analysis and is expressed as:

(1) $$k=A\cdot \exp\left(-\frac{E_{a}}{R\cdot T}\right),$$

where k, A, $E_{a}$, R and T are the rate underlying the degradation, the material constant, the activation energy, the universal gas constant and the absolute temperature, respectively.

For tensile and compressive stress–relaxation, at a chosen level of degradation (i.e., sealing force loss), the corresponding ageing times are plotted in a logarithmic diagram versus the inverse of their test temperatures. The plots are known as Arrhenius diagrams [8], and when a linear relationship is obtained, the slope represents the activation energy of the underlaying degradation process. Another approach to determine the degradation activation energy is the time-temperature superposition method [9], where stress–relaxation data determined at different test temperatures are horizontally shifted to a reference stress–relaxation curve of a chosen reference temperature, in order to construct a master curve. As for the first method, the shift factors are plotted in an Arrhenius diagram versus the inverse of temperature, and the activation energy of the underlaying degradation process is obtained from the resulting slope. Another well-known method in chemistry but less utilized in mechanical testing is the use of integrated laws based on kinetic studies [10,11]. So far, the congruency of kinetic studies based on stress relaxation data to the Arrhenius treatment were only tested on foamed poly-siloxane rubber [12]. All these three methods, namely, the Arrhenius approach, the time-temperature superposition technique and the kinetic study, are based on Arrhenius treatment. However, through analyzing the shift factor of oxygen consumption rate in comparison to the shift factors of elongation at break and compression stress drop to 90%, studies have shown that material degradation often does not follow an Arrhenius temperature dependence [13,14,15]. The curvature in Arrhenius diagrams may be due to many factors. The first factor is heterogeneous ageing, which is due to diffusion limited oxidation (DLO) effects [16]. DLO appears when the rate of oxygen consumption is higher than oxygen diffusion in the material. As a result, the progress rate of the ageing will be reduced, and the material will age heterogeneously. The second factor is the Arrhenius relationship definition (Equation (1)), which is based on the assumption that the degradation involves a single thermally activated chemical process with a rate k. Different degradation processes at high and low temperature ranges with different activation energies have been established [14]. So far, the competing processes were investigated only at low and high temperature regimes [14], and no mathematically based kinetic modeling description evolving a total apparent activation energy over the exposure time of these processes within an appropriate temperature range was considered. Another factor that may result in the curvature is when the collected data does not sufficiently cover an appropriate time frame and temperature range necessary for the evolution of the degradation of the chosen property [13]. At lower ageing temperatures and in the time frame of the conducted experiments, which could be relatively long, smaller degradation rates may distort the extrapolation for the lifetime prediction analysis. Thus, the extrapolation should be handled with caution, and all influencing factors should be eliminated [17].

During stress relaxation measurements, both physical and chemical processes contribute to the reduction of stress. Although chemical ageing is the dominant effect at high temperature and long ageing time, physical relaxation should not be neglected, as it contributes to more than 10% force drop [18]. For applications at moderate temperatures, this contribution could represent the main part of the overall relaxation, even for long times of use. To overcome the smaller degradation rates issues that may distort the lifetime extrapolation, two solutions are possible. The first one is to perform long-term measurements [19]. The second one, in order to avoid the long times necessary for performing long-term measurements, is to simulate the material behavior during extended ageing times.

Regarding the modeling of elastomers that undergo microstructural changes, i.e., chain scission and crosslinking, very interesting constitutive theories based on the two-network chain theory first introduced by Tobolsky [20,21,22] and Andrews et al. [23], ranging from phenomenological to physically motivated approaches, were developed [24,25,26,27,28]. The theory relies on the coexistence of a degenerating and newly created network. An extension to the multiple network theory was proposed in [29]. Motivated by the two-network theory, three dimensional constitutive models ranging from homogeneous and non-homogeneous deformation induced scission to thermally induced scission have been developed and are discussed in a review article of Wineman [30]. However, modeling the reversible physical relaxation during ageing has been omitted in the major part of the conducted studies, except in [29]. It is worth mentioning that an approach to estimate the long-term stress relaxation of natural rubber (NR) using the spectral analysis to separate the relaxation processes was developed by Ronan et al. [31,32]. In [31,32], two chemical relaxation processes were determined, and the summation of the separated processes enabled to recalculate a fitting curve of the compression stress–relaxation. However, no validation of the prediction was given, and a systematic deviation of all fits from the experiments was observed. In these models, mostly no mathematically based kinetic modeling description of the decay functions for lifetime estimations was given, except in a recent publication [33].

The motivation of our work is to implement constitutive equations into a finite element (FE) code under finite strain framework, covering the reversible and irreversible relaxation stages of elastomers during ageing, with extended time frames suitable for the simulation. The present investigation is based on the continuous stress relaxation analysis. Only chain scission evolution is taken into account in the modeling, as newly formed crosslinks in compression state during ageing do not contribute to the reduction of stress according to the two-network theory. The proposed thermodynamic potential is an extension of the existing ageing models [24,25,29]. Based on our previous investigations [18], an identification strategy was adopted according to the determined characteristic times related to the degradation processes of the investigated samples. The separation of physical and chemical relaxation stages was obtained through the analysis of compression set (CS) measurements after tempering, in combination with the compression stress relaxation of ethylene propylene diene (EPDM) O-ring samples compressed by 25%. After separation of physical and chemical relaxation, two degradative processes for chemical relaxation at low and high exposure times, represented by two decay functions, are identified.

Computer simulations are compared and validated with experimental results. The analyses were carried out on DLO free samples, which were investigated and described in previous works [16,34], and the integrated rate law was applied to the experimental results to test the applicability of the kinetic study.

Lifetime estimations of the simulated and the experimental results were discussed based on the following three approaches:

1. the Arrhenius treatment
2. the time-temperature superposition technique
3. the kinetic study

The validation of the simulation results is performed through the comparison of the determined activation energies resulting from the experiments and the simulations with the three approaches. The evolution of the two degradative competing processes during ageing with two reaction rates is also investigated in this paper. A temperature-independent and a degradation rate-based mathematical model that evolve a total apparent activation energy over the exposure time is proposed. The contribution of each process to the total apparent activation energy is determined. This gives an insight into the resulting degradation history and may clarify the presence of the curvature in the Arrhenius plots due to insufficient experimental data where only one degradative process is activated. The curvature will result in different activation energies, even for homogeneously but not sufficiently long aged samples. This would distort the extrapolation to a lower temperature. The main advantage of the presented approach which distinguishes physical and chemical processes is the possibility to quickly validate the interpolation at lower temperatures within the range of slower chemical processes without forcing a straight-line extrapolation.

## 2. Materials and Methods

### 2.1. Materials

Commercial EPDM O-rings were used for the experiments. The provided O-rings had a cord diameter of 10 mm and an inner diameter of 190 mm. The base rubber had an ethylene content of 48 wt.% and 4.1% ethylidene norbornene (ENB). The EPDM compound was peroxide-cured and contained 90 phr carbon black fillers, and no plasticizer.

### 2.2. Methods

#### 2.2.1. Continuous Stress Relaxation Experiments

Continuous compression stress relaxation experiments were carried out on O-ring segments each with a length of 40 mm. Three O-ring segments were investigated per test temperature, using EB 02 rigs in an EB 22 oven, both from Elastocon (Brämhult, Sweden). O-ring segments were age-compressed by 25% at four different temperatures (75 °C, 100 °C, 125 °C and 150 °C). Details on the thermomechanical sample conditioning before the experiments and the monitoring of isothermal force measurements during the experiments have already been reported in our previous publications [16,18,34]. The isothermal experimental results are presented as the normalized continuous stress relaxation $R_{c}$ which is defined as the ratio of the continuously measured force to the initial force for compression. The median curves of three measurements are presented in the following.

#### 2.2.2. Compression Set

Half O-rings were aged in ovens compressed by 25% between steel plates for a given ageing time. After disassembly at room temperature, the samples were allowed to recover for 5 days at room temperature, and were then placed again in an oven for one day at 100 °C to accelerate the recovery towards equilibrium [35]. It has been established in our previous investigation [18] that the measured CS after tempering presents a quasi-equilibrium value and reflects only the irreversible chemical reactions. The recovered height of O-ring segments, after tempering for one day by 100 °C, was measured at 7 to 10 positions and CS, with an error of measurement of about ±3% calculated from the average value.

## 3. Modeling

The adopted constitutive model in this study is formulated within the framework of large deformation hyper-elastic quasi-uncompressible materials. The aim is to develop a model that takes into account the reversible physical relaxation as well as the irreversible chemical relaxation. The discretization time (ageing time) for each ageing temperature will be chosen long enough to estimate the long-term behavior of the material. The multiplicative decomposition of the deformation gradient F (Equations (2) and (3)) at a given temperature into dilatational and distortional parts is adopted. It is to mention that the thermal decomposition of the deformation gradient F is beyond the scope of the present modeling, since the experiments were performed under isothermal conditions, and no ageing-induced volume change is considered. Moreover, as the imposed deformation according to the experiments is set to 25%, no deformation induced scission is taken into account in the present modeling, as our comparison between the effect of compressed and uncompressed ageing did not indicate differences between the samples [36]. However, deformation combined with temperature could affect scission in special cases where the deformation in tension exceeds 200%, as has been reported in the literature [20,37].

(2) $$F=\bar{F}.F^{vol},$$

(3) F¯=J−13.F,

here, J is the transformation jacobian. The left Cauchy green tensor B is also written in term of the multiplicative decomposition (Equation (4))

(4) $$B=\bar{B}.B^{vol},$$

The strain energy function is split into a deviatoric and a volumetric part according to Equation (5):

(5) $$\Psi \left(B\right)=\bar{\Psi }\left(B\right)+\Psi^{vol}\left(J\right),$$

The neo-hook potential (Equation (6)) is adopted, since it represents the Helmholtz free energy of a molecular network with Gaussian chain-length distribution and has proven its validity when a material undergoes microstructural changes, as long as the temperature and the deformation histories have been homogeneous [38].

(6) $$\Psi \left(B\right)=C.\left(tr\left(\bar{B}\right)-3\right)+\frac{1}{D}\;{\left(J-1\right)}^{2},$$

where $C=\frac{1}{2}n\left(t\right)\cdot k\cdot T$.

n(t) is the effective number of crosslinks as a function of exposure time, k is Boltzmann’s constant and T is the absolute temperature.

### 3.1. Physical Relaxation

The viscoelastic short-term behavior commonly called physical relaxation is a reversible process which occurs at short times during the continuous stress relaxation experiment. This relaxation process is due to chain rearrangements under the compression load. However, in the case of quasi-static mechanical response of elastomers and their inelastic response such as Mullin’s effect and permanent set, permanent damage should be considered, as the process is irreversible [39,40]. 

For hyper-elastic material behavior, the relaxation coefficients in ABAQUS^®^ [41] (Equation (7)) are applied to the constant C (Equation (6)). The time domain relaxation response, in a standard static analysis and under isothermal conditions, corresponds to an elastic solution based on instantaneous elastic moduli.

(7) CR(t)=C0·(1−∑k=1ng¯kp·(1−exp(−tτk)),

where ${\bar{g}}_{k}{}^{p}$ and $\tau_{k}$ are the Prony series parameters and $C^{0}=\frac{1}{2}n\left(0\right)\cdot k\cdot T$.

### 3.2. Chemical Ageing

With a constant strained condition at a certain time, part of the polymer chains that have achieved the maximum allowable strain at a given temperature will fail. The effective number of crosslinks could depend on deformation, temperature history and time. Due to the simplifying assumptions presented at the beginning of Section 3, the effective number of crosslinks should be chosen as a decreasing function of t. One can suppose the following dependency (Equation (8)) according to the Tobolsky two-network theory [23]:

(8) $$\dot{n}\left(t\right)=\frac{dn\left(t\right)}{dt}=-\frac{1}{\tau }n\left(t\right),$$

The solution of the first order kinetic reaction is:

(9) n(t)=n0.exp(−tτ),

here, $n_{0}$ is the initial crosslink density at t=0 and τ is the retardation time, which is strongly dependent on the ageing temperature [29]. The Cauchy stress is given by Equation (10).

(10) σ(t)=n0.exp(−tτ).k.TJ(B¯−13 tr(B¯).1)+2D(J−1).1,

Hence, the normalized continuous stress relaxation $R_{c}$ following the Tobolsky two-network theory is expressed in Equation (11)

(11) $$R_{c}=\frac{\sigma \left(t\right)}{\sigma \left(0\right)}=\frac{n\left(t\right)}{n\left(0\right)},$$

It is to be mentioned that the chosen thermodynamic potential, with its actual state variables and parameters, is only valid for homogeneously aged samples. However, an extension to take into account heterogeneous ageing may be done through considering the diffusion reaction equation [42]. Furthermore, through the multiplicative decomposition of the deformation gradient, a possibility to model damage and/or ageing induced volume change, where the shrinkage in volume leads to an increase of the stress during tensile stress relaxation, which is straightforward and feasible [43,44], but not applied here.

## 4. Results and Discussion

### 4.1. Continuous Stress Relaxation

**Figure 1:**
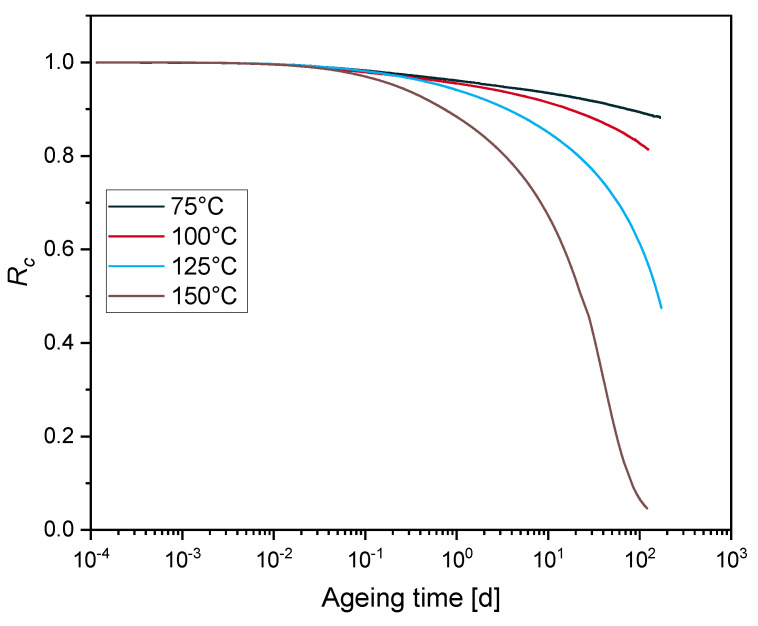
EPDM normalized continuous stress relaxation (*R_c_*) data at different temperatures.

Figure 1 shows the experimental compression stress relaxation results of EPDM aged at 75 °C, 100 °C, 125 °C and 150 °C over ageing time.

$R_{c}$ exhibits two regions: a flat decrease at the beginning and a stronger decrease at longer ageing times. The first phase correlates with physical relaxation, while the second phase correlates with chemical relaxation [45]. The second phase is more pronounced for EPDM aged at 125 °C and 150 °C. For EPDM aged at 75 °C and 100 °C, the samples are not sufficiently aged to obtain the second phase as the minimum $R_{c}$ values are 0.88 and 0.8, respectively, although the experiment was run for over 168 days and 124 days.

### 4.2. Kinetic Study

In this section, a kinetic study based on the experimental results displayed in Figure 1 is performed, in order to determine the reaction order of the degradation. An *n*^th^ order rate equation is expressed as:

(12) $$\frac{d\left[R_{c}\right]}{dt}=k_{n}\cdot {\left[R_{c}\right]}^{n},$$

where $k_{n}$ is the *n*^th^ order rate constant, $R_{c}$ is the ratio of the continuously measured force to the initial force for compression, *t* is the ageing time and n is the order of reaction.

The integrated rate law of Equation (12) is:

(13) $$\{\begin{array}{ll} \frac{1}{{\left[R_{c}\right]}^{n-1}}=\left(n-1\right)\cdot k_{n}\cdot t+\frac{1}{{\left[R_{c}\right]}_{0}{}^{n-1}} & for\;n\neq 1\\ \ln\left(\frac{{\left[R_{c}\right]}_{0}}{\left[R_{c}\right]}\right)=k_{1}\cdot t & for\;n=1 \end{array},$$

where ${\left[R_{c}\right]}_{0}$ is the initial normalized force.

**Figure 2:**
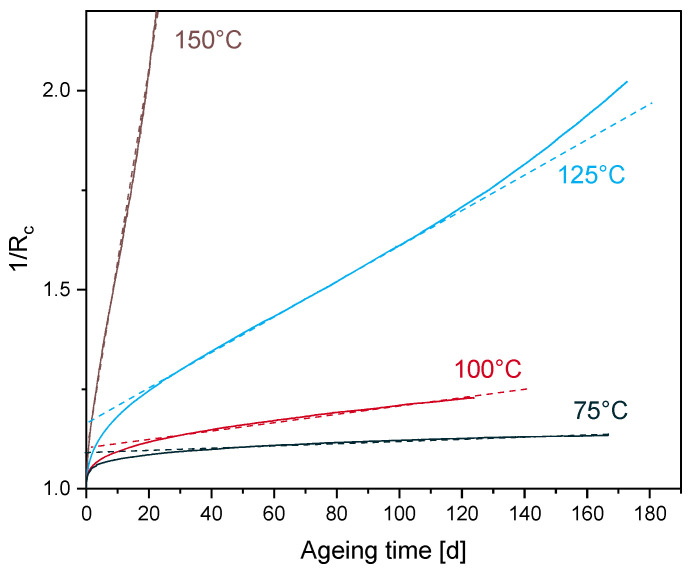
Integrated second order rate plots of EPDM relaxation data at different temperatures (experiment: solid lines, fit: dashed lines).

Following the investigation of Patel et al. [12], the stress relaxation data were analyzed according to the integrated rate law, and only the best fit is presented in this study. Figure 2 presents the integrated second order function of the experiments and the relative fits. For EPDM aged at 125 °C and 150 °C, the best fits are obtained until 115 d and 24 d, which correspond to the $R_{c}$ values of 0.6 and 0.49, respectively.

**Figure 3:**
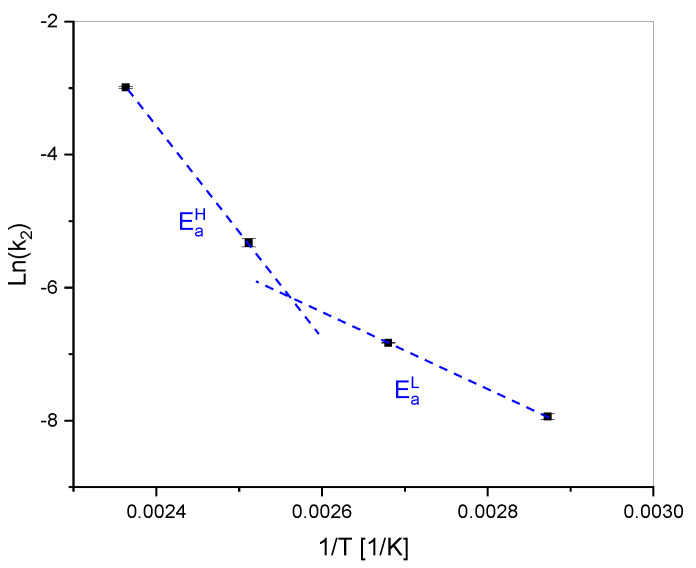
Correlation of the second-order kinetic rate constants $k_{2}$ to the Arrhenius relationship. (linear regression fit for 150 °C + 125 °C and 100 °C + 75 °C, respectively: blue dashed lines).

For EPDM aged at 125 °C, 100 °C and 75 °C, the linear portion has started for $R_{c}$ values of 0.8, 0.9 and 0.93, respectively. From the kinetic study, the stress relaxation data follows a second order kinetic behavior. Each second-order rate constant $k_{2}$ derived from the slope of the dashed lines in Figure 2 is determined to test the correlation with the Arrhenius relationship depicted in Figure 3 by drawing an Ln-plot of $k_{2}$ versus 1/T.

Although the experimental results show a good agreement with the second order kinetics, a poor adherence with the Arrhenius relationship is obtained from Figure 3. From this observation, it is not clear whether the curvature is due to the lack of data over an appropriate time frame, or if it involves two activation energies, one for low temperature regimes ($E_{a}^{L}$) and one for high temperature regimes ($E_{a}^{H}$) [14], as schematically drawn in Figure 3 (blue dashed lines $E_{a}^{L}$ = 50 kJ/mol; $E_{a}^{H}$ = 131 kJ/mol), with a crossover temperature of 117 °C. Although a non-Arrhenius behavior for EPDM was reported at low ageing temperatures below 80 °C [46], it should be noted that the over-compression method was used in this investigation. The over-compression method consists on mechanically over-straining the sample for a short time period before returning to application strain in order to obtain a quasi-equilibrium sealing force. Another non-Arrhenius behavior was established for another EPDM [14], through the combination of elongation at break and oxygen consumption rate data in an Arrhenius plot. Two activation energies for low and high temperature regimes $E_{a}^{L}$ = 78 kJ/mol and $E_{a}^{H}$ = 127 kJ/mol, respectively, were identified with a crossover temperature of 123 °C. However, the estimation of the second-order rate constants $k_{2}$ in Figure 2 was not calculated at the same scale of $1/R_{c}$ for all ageing temperatures. The same issue was also encountered in the conducted investigation of Patel et al. [12] on foamed poly-siloxane rubber at ageing temperatures below 120 °C. This is due to the lower values of $1/R_{c}$. From Figure 2, the maximum value of $1/R_{c}$ at 75 °C is 1.13 and the linear portion starts from 1.07. The linear fit for all temperatures was conducted between 1.07 and 1.13 (not presented in this paper) and a poor fit to the linear regression was obtained for 150 °C and 125 °C aged samples. Within the fit interval, the maximum ageing times of samples aged at 150 °C and 125 °C are 1 d and 7 d, respectively. The impact of the degradation rate on the $k_{2}$ values and consequently on the obtained curvature in Figure 3 will be checked in the following sections, in which longer ageing times from a simulation will be adopted, in order to overcome the possible lack of experimental data at low temperatures. The modeled estimations of the long-term stress relaxation will then serve to retest the validity of the kinetic approach to the Arrhenius relationship.

### 4.3. FE Simulation Results

**Figure 4:**
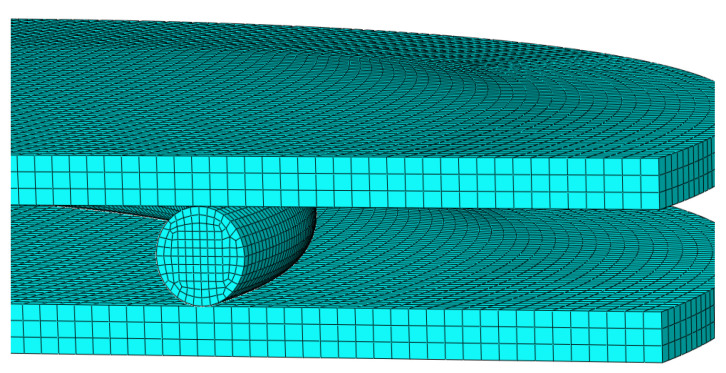
Meshed O-ring segment in assembly between two steel plates (Young´s modulus: E = 210 GPa, Poisson’s ratio: ν = 0.3).

The constitutive model is implemented as a user-defined material behavior in a standard large displacement based large strain finite element code ABAQUS/Standard^®^ (version 6.14-6, Dassault Systemes Simulia Corp., Providence, RI, USA) [41], via a user subroutine UMAT. An O-ring segment with the same dimensions as in the experiments was placed between two steel plates and meshed with 3D elements C3D8H (8-node linear hybrid bricks with constant pressure), as shown in Figure 4. For the simulation, the seal was compressed by 25%, which is in agreement with the compression for the experimental investigations. The constitutive equations are considered at the Gauss point of each finite element. In the following, the simulation results are expressed as the ratio $R_{c}$ versus the chosen ageing time necessary for the simulation. From Equation (11), $R_{c}$ values are directly computed in the UMAT and stored in ABAQUS/Standard^®^ in an array of solution dependent state variable STATEV.

**Figure 5:**
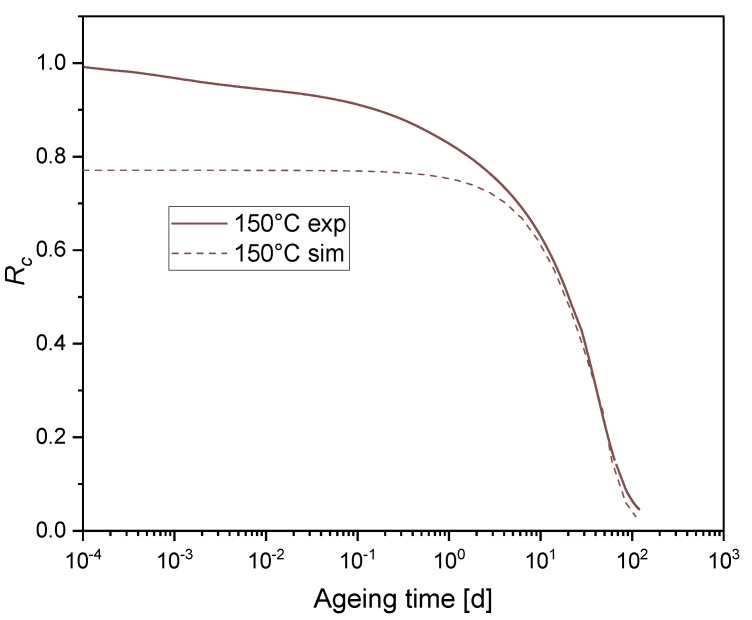
Normalized continuous stress relaxation for EPDM aged at 150 °C: Experiment and simulation with one degradative process and a relaxation time τ= 20 d.

Figure 5 shows the experimental and the simulation results of the normalized continuous stress relaxation $R_{c}$ for EPDM aged at 150 °C.

**Figure 6:**
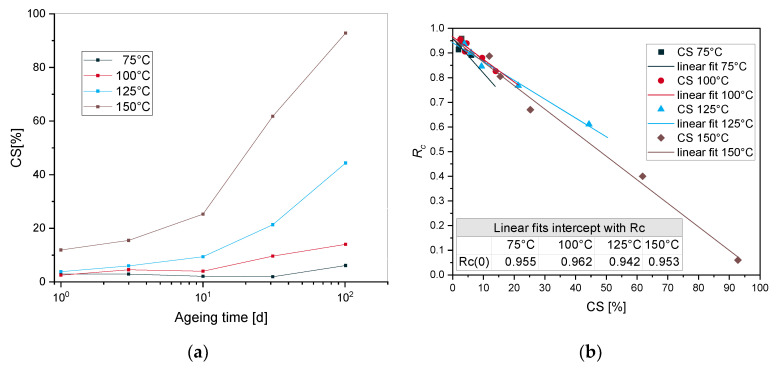
(**a**) CS after tempering versus ageing time for EPDM at different temperatures, (**b**) Initiation times at different temperatures.

It was expected that the chosen Maxwellian stress relaxation fits well with the experimental data at a sufficiently long time (starting from t > 10 d) but not at short and intermediate times. This is due to the Maxwell system of Equation (9). In fact, when applying an $Ln\left(R_{c}\right)$ to the experimental data and considering Equation (11), a linear relationship is to be expected, which is not the case. To better fit the experimental data at short and intermediate times, an extension of the scission relaxation function based on the model of Shaw et al. [29] is proposed. For a constant temperature history, a chosen number of first order chemical relaxation processes take place simultaneously, so that the solution of the kinetic reaction of scission is shown in Equation (14):

(14) n(t)=n0.∑i=1nAiexp(−tτi), Ai≥0 and ∑i=1nAi=1,

CS data after tempering are used in the identification to separate physical and chemical relaxation stages. Figure 6a shows the evolution of equilibrium CS versus ageing time for EPDM aged at 75 °C, 100 °C, 125 °C and 150 °C. According to our previous investigations [18], characteristic times related to the degradation processes were determined for EPDM aged at 125 °C. The chemical initiation time which represents the end of physical relaxation stage and the onset of chemical relaxation one [18] was determined through the intercept of *R_c_* versus CS values after tempering at CS = 0. With the *R_c_* intercept values presented in Figure 6b, one can determine the chemical initiation times at 150 °C, 100 °C and 75 °C, that are suitable for the modeling section from the stress relaxation data versus ageing times (Figure 1).

The initiation time values of EPDM aged at 150 °C, 125 °C, 100 °C and 75 °C are collected in Table 1. It was expected that the initiation time of EPDM aged at 75 °C would be higher than that determined in Table 1 as, normally, the initiation time increases with decreasing temperatures. This is probably due to the lower values of CS after tempering. Even for samples aged at 75 °C for five years, CS did not exceed 14% [36].

**Figure 7:**
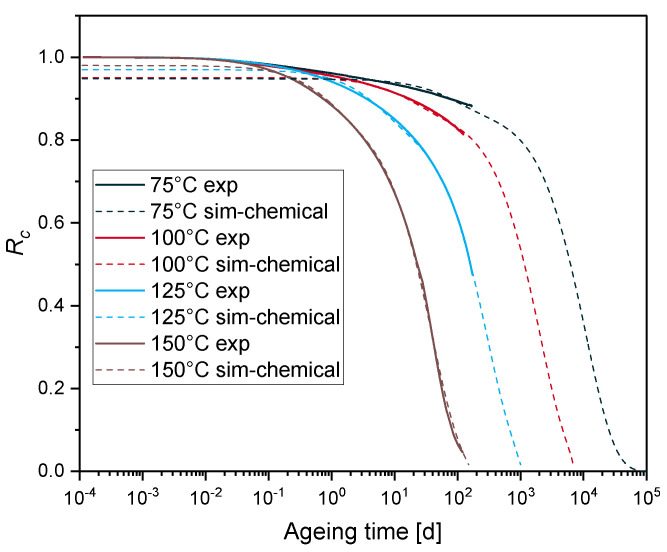
Normalized continuous stress relaxation for EPDM aged at 75 °C, 100 °C, 125 °C and 150 °C (experiment: solid lines, simulation with two first order chemical relaxation processes: dashed lines).

Figure 7 presents the normalized continuous stress relaxation $R_{c}$ with two first order chemical relaxation processes. Three first order chemical relaxation processes were also tested (not presented in this paper), and no noticeable difference compared to the two first order chemical relaxation processes was observed. Thus, we limited ourselves to two first order chemical relaxation processes. The material parameters used in this simulation are summarized in Table 2, in the columns relating to the chemical relaxation parameters. Compared to Figure 5, Figure 7 shows better fits of the experimental results, at intermediate and long times.

To identify the physical relaxation parameters, the experimental stress relaxation data for EPDM at different ageing temperatures until the initiation times (Table 1) are introduced in ABAQUS^®^. An internal tool identifies the Prony parameters until the given initiation times. A maximum number of terms N = 13 of the Prony series was first set in ABAQUS^®^. The internal tool performs the least-squares fit from N = 1 to N = 13, until convergence is achieved for the lowest N, with respect to the error tolerance, which is set equal to 10^−5^. The convergence was reached for N = 2 for EPDM aged at 150 °C, 125 °C and 75 °C. For EPDM aged at 100 °C, only one Prony series term was needed for the convergence. The resulting parameters are presented in Table 2 in the columns relating to the physical relaxation parameters.

**Figure 8:**
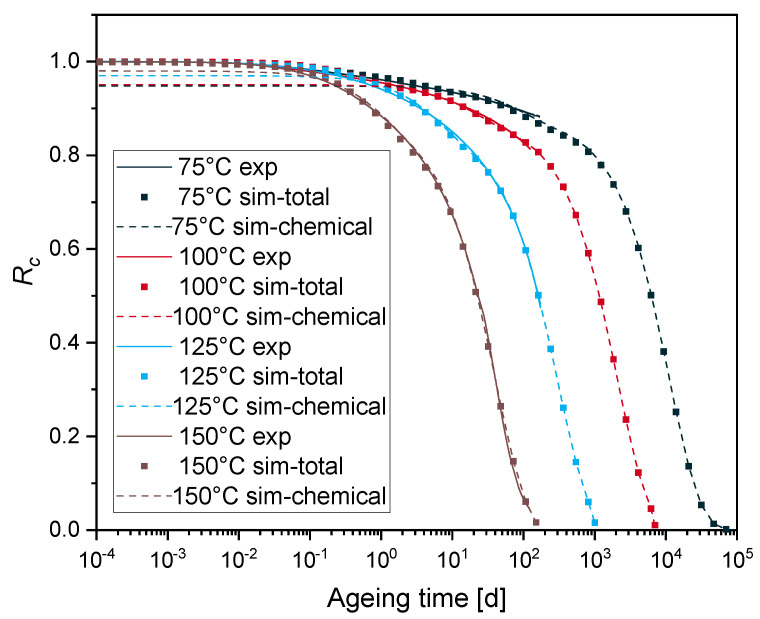
Normalized continuous stress relaxation for EPDM aged at 75 °C, 100 °C, 125 °C and 150 °C (experiment: solid line, simulation with two first order chemical relaxation processes: dashed lines, simulation of the normalized total stress relaxation: points).

Figure 8 shows the simulation results of the normalized continuous stress relaxation at different ageing temperatures with physical relaxation and chemical ageing, which are labelled with “sim-total” in the legend. The set of material parameters used in the simulation is summarized in Table 2 (physical relaxation parameters and chemical relaxation parameters).

### 4.4. Validation of the Model

The validation and the discussion in this section will involve three different treatments, namely, the Arrhenius treatment, the time-temperature superposition technique and the kinetic study. The resulting apparent activation energy with each method will be used to validate the numerical model. Any activation energy resulting from different experimental procedures/tests will not be suitable to serve as a base to validate our results. Indeed, when considering different tests (e.g., compression set and elongation at break), even with the same geometry of samples that have aged homogeneously, different activation energies are to be expected [47].

#### 4.4.1. Time-Temperature Superposition

The main advantage of the time-temperature superposition technique (TTS) compared to the Arrhenius treatment is the fact that all time-dependent data generated at each ageing temperature are used in the TTS, while only one data point from each data set at a given criterion (percent of degradation of a chosen property) is used in the Arrhenius approach.

**Figure 9:**
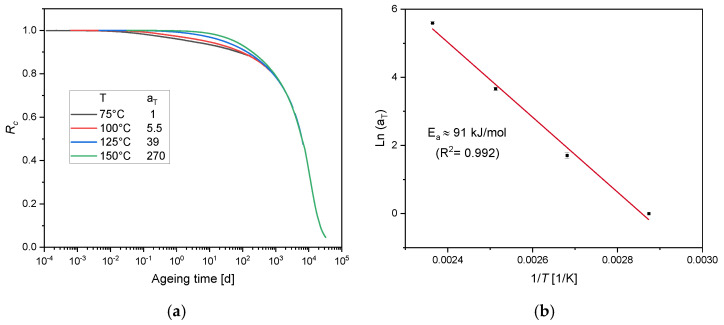
(**a**) TTS of *R_C_* at the four different temperatures with the reference temperature of 75 °C and corresponding shift factors. (**b**) Arrhenius plot of shift factors a_T_.

TTS is applied to the experimental data displayed in Figure 1. The measured data of $R_{c}$ at 100 °C, 125 °C and 150 °C are shifted along the logarithmic time scale, until superposition with the reference temperature 75 °C is achieved, as shown in Figure 9a. The available experimental data at 75 °C should be handled with care in this analysis, due to the smaller degree of degradation of the material (maximum sealing force loss ≃ 10%).

For this purpose, we first shifted the 100 °C curve longer ageing times, until a superposition was obtained at the last part with the 75 °C data. For 125 °C and 150 °C data, the superposition was better, as seen in Figure 9a. To determine the functional relationship between the shift factors and the temperature, the Arrhenius treatment is combined with TTS (Figure 9b), through drawing the shift factors (a_T_) in an Arrhenius diagram.

From Figure 9b, a linear relationship is obtained, and the apparent activation energy is *E_a_* ≃ 91 kJ/mol.

#### 4.4.2. Arrhenius Approach

**Figure 10:**
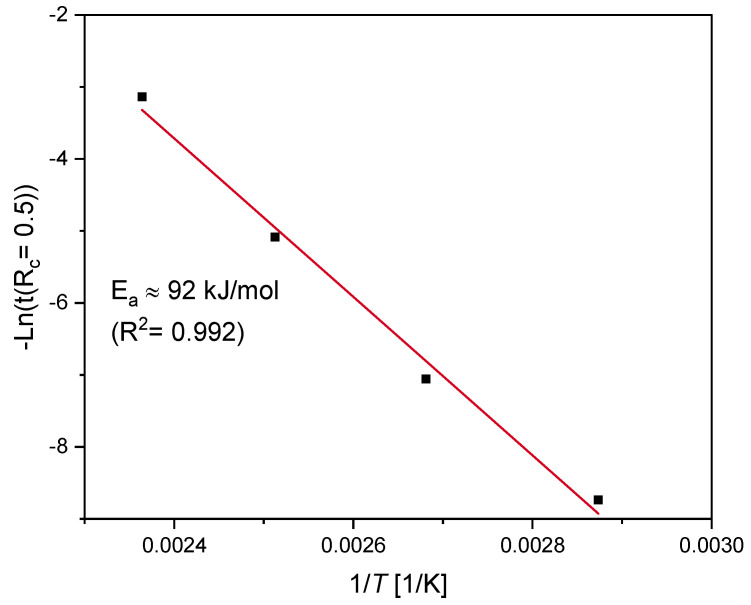
The Arrhenius plot for the relaxation half-times t (*R_c_* = 0.5).

The FE simulation results (Figure 8) with the identified parameters (Table 2) are in good agreement with the conducted experiments. In order to validate the prediction of the model especially at the lower ageing temperatures of 100 °C and 75 °C, at which the experimental sealing force loss only reaches 20% and 12%, respectively, the Arrhenius approach is adopted. This enables the determination of the activation energy and therefore to predict the lifetime of EPDM seals. But it is valid only when the degradative ageing of the material is homogeneous, i.e., free of DLO effects [13]. Previously, intender modulus measurements were carried out on the surface layers of aged EPDM O-ring surfaces in uncompressed as well as in compressed state by 25%, with the same material compound and geometry [16,34,36,48]. The samples aged at 150 °C age homogeneously until 101 days (which corresponds to *R_c_* = 0.06 from the experimental results, see Figure 1); as for these samples, no heterogeneous hardening was observed [16,34]. The ageing times at 50% force drop (*R_c_* = 0.5) are determined from the simulation results given in Figure 8. By plotting the relaxation half-times t (*R_c_* = 0.5) versus 1/*T* in an Arrhenius diagram (see Figure 10), a linear relationship is obtained, and the activation energy for 50% force drop is determined as $E_{a}≃$ 92 kJ/mol. This value is very close to the value obtained from the experimental results with the TTS (≃ 91 kJ/mol, see Figure 9).

#### 4.4.3. Kinetic Treatment

**Figure 11:**
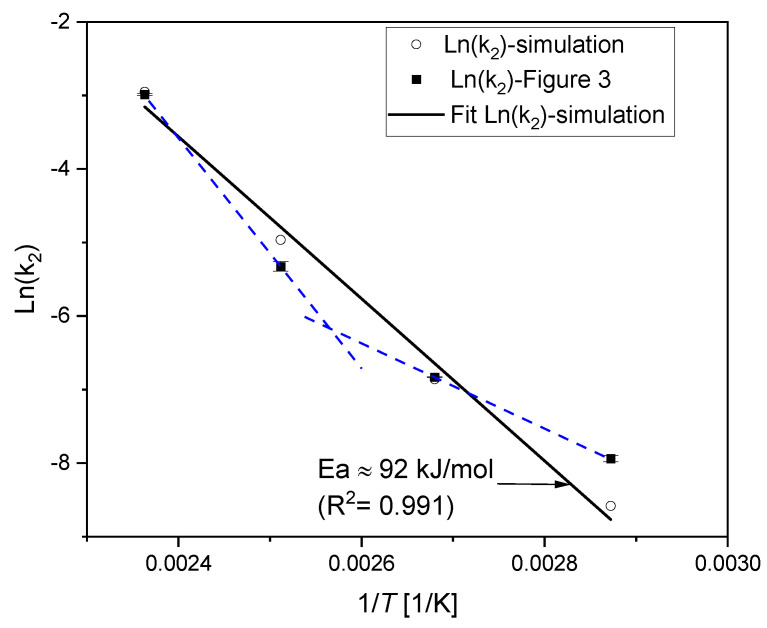
The correlation of the second-order kinetic rate constants to the Arrhenius relationship: Comparison of the simulated rates with the values of Figure 3.

The same procedure presented in the kinetic study section was reproduced with the modeled estimations of the long-term stress relaxation, in order to test the validity of the Arrhenius approach. From Figure 6b, the chemical initiation time for *R_c_* is around 0.94, which represents the onset of irreversible chemical degradation. Hence, it would be suitable to perform the analysis on sufficiently aged samples at the same degree of degradation for all ageing temperatures. All fits are performed for the simulation results of *R_c_* sections between 0.8 and 0.26, and the resulting slopes ($k_{2}$) are determined. The correlation of the $k_{2}$ to the Arrhenius relationship is shown in Figure 11. A better fit to the Arrhenius approach for the simulated results with an *E_a_* ≃ 92 kJ/mol is obtained. The obtained *E_a_* value with the kinetic study that was performed on the simulation results over an extended time frame is the same as that for 50% loss of sealing force, which was determined via the Arrhenius approach shown in Figure 10. The *E_a_* value with the kinetic study, in comparison with the TTS applied to the experimental data given in Figure 9, is very close.

From the three methods, similar values of activation energies are obtained, which demonstrates the validity of the numerical model used in the previous section. This confirms that the curvature in the Arrhenius analysis could arise from the fact that the degradation rate of the mechanical property (e.g., *R_c_*) is too slow within the available experimental ageing times. Considering the experimental relaxation data (Figure 1), especially for the low ageing temperatures and the chemical relaxation time values in Table 2, a closer look to the contribution of different chemical processes to the overall relaxation is noteworthy. This could be done through analyzing the different activation energies that are the subject of the following section.

### 4.5. Contribution of Different Chemical Processes

Examining the temperature dependency at a given force decrease by using the simulation results presented in Figure 8, one can determine the activation energy with the Arrhenius relationship. In fact, a lifetime criterion which has to be reached at every ageing temperature is used for the Arrhenius relationship. For instance, an $R_{c}$-based criterion is chosen. The logarithm of the times to reach the criterion can then be plotted in an Arrhenius diagram vs. the inverse temperature. A straight line was obtained at each $R_{c}$. Table 3 shows the total activation energy $E_{a}^{total}$ at different $R_{c}$ calculated from the slope with the Arrhenius relationship (ln (ageing time) vs. 1/T), and its respective regression coefficient.

Good correlations to the Arrhenius approach, especially for *R_c_* values lower than 0.95, are obtained and evidenced by high coefficients of regression (Table 3).

**Figure 12:**
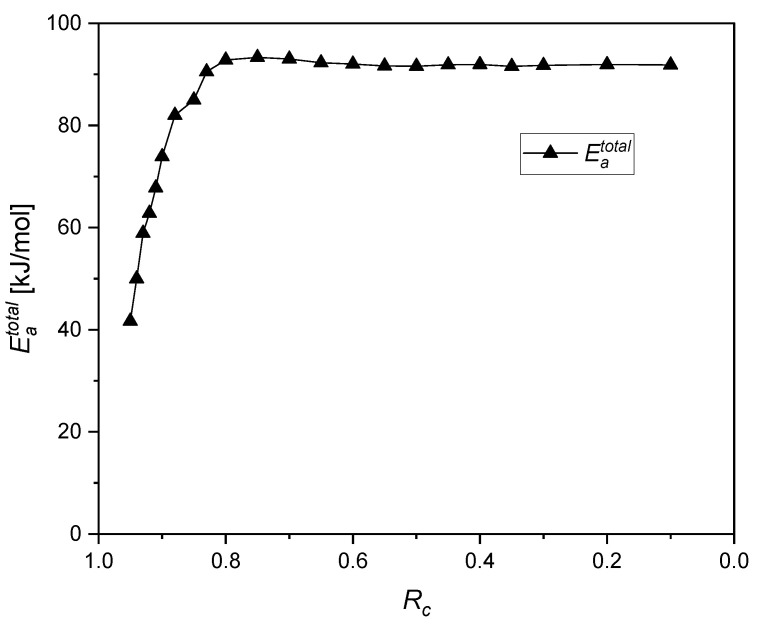
The evolution of the total activation energy $E_{a}^{total}$ as a function of the normalized continuous stress relaxation $R_{c}$.

Taking the values from Table 3, the evolution of $E_{a}^{total}$ as a function of $R_{c}$ is shown in Figure 12. One can clearly observe first an increase of $E_{a}^{total}$ until $R_{c}=0.75$, followed by a saturation or a plateau at about ≃ 92 kJ/mol.

**Figure 13:**
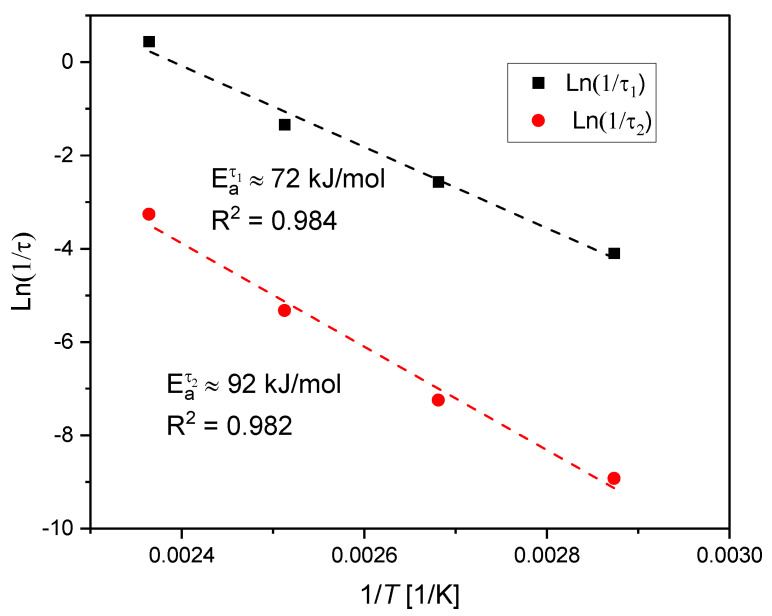
Assessment of the activation energy of each process based on relaxation times at different isothermal temperatures.

From the modeling section, two first order chemical relaxation processes with two relaxation times $\tau_{1}$ and $\tau_{2}$, respectively, were determined, each related by the Arrhenius relationship, as depicted in Figure 13. The activation energies $E_{a}^{\tau_{1}}$ and $E_{a}^{\tau_{2}}$ for the first and the second process respectively are also presented in Figure 13.

Since the Arrhenius approach is based on the assumption that the degradation involves only a single thermally activated chemical process, the resulting apparent total activation energy yields could be assumed as weight-averaged values of different activated chemical processes. The evolution of the total activation energy of the system $E_{a}^{total}$ (see Figure 12) could be connected to the single-process energies $E_{a}^{\tau_{1}}$ and $E_{a}^{\tau_{2}}$ through a rule of mixture, as presented below:

(15) $$\{\begin{array}{c} E_{a}^{total}=\alpha_{1}\cdot E_{a}^{\tau_{1}}+\alpha_{2}\cdot E_{a}^{\tau_{2}}\\ \alpha_{1}+\alpha_{2}=1\\ \alpha_{1},\alpha_{1}\geq 0 \end{array},$$

For each $R_{c}$ state and its corresponding $E_{a}^{total}$, values of the weighting factors $\alpha_{i}$ are calculated. To represent a progressive transition between the two states, the evolution of the fraction of process $\alpha_{i}$ is chosen as:

(16) $$\{\begin{array}{c} \alpha_{1}=A\cdot \left[1+\tanh\left(\frac{R_{c}-x}{l}\right)\right]\\ \alpha_{2}=A\cdot [1+\tanh\left(\frac{x-R_{c}}{l}\right)] \end{array},$$

where x is the crossover point between the $\alpha_{i}$. At this crossover point, each process contributes with $\alpha_{1}=\alpha_{2}=\frac{1}{2}$ to the overall reaction; l is a scaling factor representing the rate or velocity of decrease or increase of a process [49].

**Figure 14:**
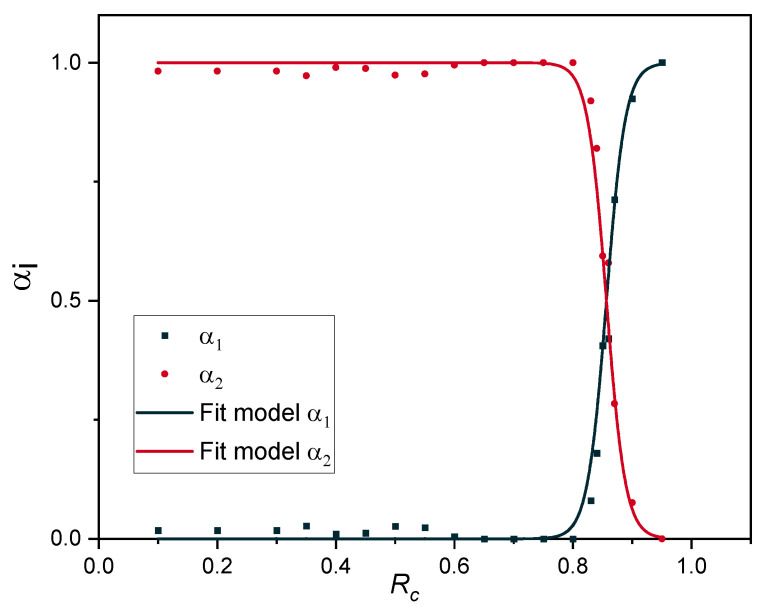
Evolution of the degradative processes.

Calculated values of $\alpha_{i}$ and the corresponding model fits are shown in Figure 14. The parameters of the model are summarized in Table 4.

The fit result is in excellent agreement with the data points, evidenced by a high coefficient of regression (Table 4).

It is worth mentioning that material compounds have an influence on several non-Arrhenius complications and that the curvature can arise from faster stabilizer consumption/migration or antioxidant interactions as solubility and evaporation effects are closely linked to temperature [50]. The investigation of non-Arrhenius behavior [13,14,15] was performed on EPDM with different compound recipe and geometry from the material of the present study. In [13,15], disk-shaped samples with 12.7 mm diameter and a 2 mm width were stacked to give a height of 6 mm that are named in the following as EPDM-sheets. As a guidance in the following, we present a comparison between our material (EPDM-rings) and the one used in [13,14,15] at the same ageing temperature of 125 °C. Taking the same ageing temperature of 125 °C and a criterion of *R_c_* = 0.1, the EPDM-sheet reaches this criterion after 162 d, while our material after ageing for 162 d has an *R_c_* of 0.5. This value is determined from the experimental data. From the simulation, the EPDM aged at 125 °C achieves *R_c_* = 0.1 after 640 d. Note that the compression stress relaxation measurements of EPDM-sheet were conducted at room temperature, and the values of the initial force for compression were determined by extrapolation to t = 0 of samples aged between 1 d and 14 d [15]. Moreover, DLO of EPDM-sheet samples aged at 125 °C was detected for an ageing time of 210 d, which is not the case for our material. In our previous investigation [34], no DLO effects were detected for EPDM O-rings aged at 125° C for 549 d, although our material is thicker, which normally enhances the appearance of DLO effects.

Gillen et al. [13,14,15] have developed a non-Arrhenius model based on the transition from a high activation energy process to a low activation energy process, as the temperature is lowered. This means that a lower activation energy chemical degradation pathway begins to dominate at low temperatures and vice versa for high temperatures. In the present model, high and low activation energies are related to the degradation rate for the studied ageing temperature range (75 °C, 100 °C, 125 °C and 150 °C). This means that the transition here is degradation rate-controlled. At lower degradation rates, the chemical degradation mechanism with the lower activation energy begins to dominate and vice versa for higher degradation rates. In the model of Gillen et al. [13,14,15] the fraction of processes $\alpha_{i}$ are a function of 1/*T*, while in the present study they are *R_c_*-dependent (Equation (15) and Equation (16)). The crossover point, x, between the $\alpha_{i}$ in the present investigation is *R_c_* = 0.856, while in Gillen et al.’s model, x = 123 °C. The activation energies in Gillen et al.’s model for low and high temperature regimes are 78 kJ/mol and 127 kJ/mol, respectively. In our model, the activation energies are related to low and high degradative processes, which are 72 kJ/mol and 92 kJ/mol, respectively.

The proposed model is a temperature-independent and degradation rate-based model, which could be used to test the validity of the Arrhenius relationship for a given temperature range. The main advantage of the proposed model is its ability to test the contribution of each process to $E_{a}^{total}$ at each $R_{c}$. Even when a curvature of the experimental results is observed with the Arrhenius treatment, as long as the relationship between the fraction of processes $\alpha_{i}$ is fulfilled, the curvature results from too short ageing times or slower ageing within the available time frame.

When we take a closer look to the crossover value (Figure 14 and Table 4), the amount of each process is the same and equal to 0.5 for *R_c_* = 0.856. On the other hand, the activation energy of the first process $E_{a}^{\tau_{1}}$ (Figure 13) is close to the value of the total activation energy $E_{a}^{total}$ at *R_c_* = 0.9 (Table 3 and Figure 12), and this process vanishes at *R_c_* = 0.7 from the mathematical model (Figure 14). All these results strengthen the finding on EPDM aged at 125 °C [18], that the physical relaxation contributes to 10% of the overall relaxation process.

It has been established from the kinetic study (Figure 2 and Figure 3) that, when the data does not cover a sufficient time frame necessary for the evolution of the degradation of a chosen property, a curvature in the Arrhenius relationship is observed. In order to cover the possible lack of experimental data at low temperatures, longer ageing times for the simulation are adopted. Kinetic analysis was applied to the simulation results (Figure 11), and a better fit to the Arrhenius relationship was obtained. It has been found from the simulation results that the ageing behavior is better described with two degradative ageing processes. This result concords with the second order reaction obtained from the kinetic study, where the overall system with the kinetic analysis yields only average values of degradation, and therefore, the system does not recognize which mechanism is taking place. Simulation results are validated through the analysis of the total activation energy with different treatments (Arrhenius, TTS and kinetic study). The different activation energies derived from the simulation results and the Arrhenius relationship (Figure 12 and Figure 13) are linked to the total activation energy of the system through a mathematical model (Figure 14). The model is a temperature-independent and degradation rate-based model that could be applicable to other experiments (e.g., compression set), as long as an evolution of the activation energy during the ageing process is detected [36]. Note that the present approach is only valid for homogeneously aged samples. Although the presence of diffusion-limited oxidation has been detected so far only in EPDM samples aged at 150 °C for 101 d which represents an $R_{c}$ = 0.06, one can suppose that, for lower ageing temperatures, samples still age homogenously within the simulated time frame, which was checked for 125 °C, where even after 549 d, no DLO was detected.

## 5. Conclusions

Through the separation of physical and chemical relaxation processes, an improved method for lifetime prediction based on the Arrhenius extrapolation was developed in this study. The modeling of physical and chemical relaxation processes allowed to cover the lack of experimental data at lower temperatures and to analyze the evolution of the irreversible chemical relaxation processes. An identification strategy for the material parameters based on the relationship between stress relaxation and equilibrium compression set was proposed. Physical and chemical relaxations were modeled with the Prony series and two decay functions, respectively.

Simulation results of compression stress relaxation were tested with Arrhenius model, TTS and kinetic study through the analysis of the resulting activation energies from these three different methods.

Additionally, it has been shown that erroneous behavior can arise from data analysis at different rates of degradation. To maximize the prediction validity, it is noteworthy to analyze the data, starting from a common basis or a criterion such as degradation rate. Hence, a degradation rate-based model for compression stress relaxation of EPDM during homogeneous ageing was developed. The model presented the contribution of two degradative processes that are determined from the two decay functions of the chemical relaxation modeling to the total degradation rate of the system.

So far, it has become clear that the process with the lower activation energy remains dominant up to *R_c_* values of about 0.85. As more data become available, the validation of the evolution of processes with the model will lead to more confident extrapolations.

## Figures and Tables

**Table 1:** Initiation time at the four ageing temperatures for EPDM.

	150 °C	125 °C	100 °C	75 °C
**t_initiation_** [d]	0.13	1	1.3	0.19

**Table 2:** Set of material parameters for physical and chemical relaxation.

		Physical Relaxation Parameters		Chemical Relaxation Parameters			
		${\bar{g}}_{k}{}^{p}$	$\tau_{k}\;[d^{-1}]$	$A_{1}$	$A_{2}$	$\tau_{1}\;[d^{-1}]$	$\tau_{2}\;[d^{-1}]$
Temperature	150 °C	0.0043	0.003	0.11	0.87	0.64	26
		0.03	0.3				
	125 °C	0.0043	0.003	0.12	0.85	3.8	205
		0.03	0.3				
	100 °C	0.05	0.31	0.08	0.871	13	1400
	75 °C	0.024	0.113	0.073	0.873	60	7500
		0.095	2.23				

**Table 3:** $E_{a}^{total}$ at different $R_{c}$ with Arrhenius treatment.

$R_{c}$	$E_{a}^{total}\;[\mathrm{kJ}/\mathrm{mol}]$	Regression Coefficient
0.95	42	0.966
0.93	59	0.989
0.9	74	0.995
0.88	83	0.997
0.85	84	0.994
0.83	91	0.998
0.8	93	0.994
0.75	93	0.992
0.7	93	0.991
0.65	92	0.991
0.6	92	0.991
0.55	92	0.991
0.5	92	0.992
0.45	92	0.991
0.4	92	0.990
0.35	92	0.990
0.3	92	0.990
0.2	92	0.995
0.1	92	0.995

**Table 4:** Fit parameters of the fraction of processes.

A	x	l	Regression Coefficient
0.5	0.856	0.032	0.997
